# Decreased antigenicity profiles of immune-escaped and drug-resistant hepatitis B surface antigen (HBsAg) double mutants

**DOI:** 10.1186/1743-422X-10-292

**Published:** 2013-09-22

**Authors:** Mingshun Zhang, Guohong Ge, Yonglin Yang, Xubing Cai, Qiang Fu, Jie Cai, Zuhu Huang

**Affiliations:** 1 Department of Infectious Disease, the First Affiliated Hospital of Nanjing Medical University, Nanjing, China; 2 Department of Microbiology and Immunology, Nanjing Medical University, Nanjing, China; 3 The Third Hospital of Zhenjiang City, Zhenjiang, China; 4 Nanjing Red Cross Blood Center, Nanjing, China

**Keywords:** HBV, HBsAg, Mutation, Antigenicity, ELISA

## Abstract

**Background:**

Selective pressure from either the immune response or the use of nucleoside analogs in antiviral therapy could be driving the emergence of HBV mutants. Because of the overlap of the open reading frame (ORF) *S* for the HBsAg and ORF *P* for viral polymerase, rtM204I and rtM204V mutations in the polymerase would produce sI195M and sW196S in the HBsAg. The combined effects of immune-escaped mutations (sT118M, sG145K, sG145R) and drug-resistant mutations (rtM204I, rtM204V) on the antigenicity profiles of HBsAg has not been widely explored.

**Methods:**

To determine the combined effects of immune-escaped and drug-resistant mutants on the antigenicity profiles of HBsAg, recombinant plasmids encoding HBsAg double mutants were constructed using site-directed mutagenesis. The supernatant from each plasmid transfection was analyzed for HBsAg in the western-blotting and five of the most commonly used commercial ELISA kits in China.

**Results:**

Western-blotting assay showed the successful expression of each HBsAg mutant. All five ELISA kits manifested similar avidity, which were demonstrated by the slope of the curves, for the sT118M mutant, and sT118M-rtM204I (sT118M-sI195M) and sT118M-rtM204V (sT118M-sW196S) double mutants, suggesting that drug-resistant YMDD mutants caused negligible losses in the antigenicity of immune-escaped sT118M HBsAg. In contrast, the presence of the rtM204I (sI195M) mutation, but not rtM204V (sW196S) in combination with the sG145K mutation significantly reduced the avidity of sG145K HBsAg. The rtM204I (sI195M) mutation also decreased the antigenicity profiles for sG145R HBsAg.

**Conclusions:**

Drug-resistant mutations rtM204I (sI195M) and rtM204V (sW196S) caused significant reduction in antigenicity for the immune-escaped HBsAg mutants sG145K and sG145R, which may hamper HBV diagnosis and disease control from HBV blood-transfusion transmissions in China. The development of ELISA kits with a greater sensitivity for drug-resistant and immune-escaped HBsAg warrants further consideration.

## Introduction

The implementation of the Hepatitis B Immunization program in China resulted in a decrease in the incidence of HBV infections, from approximately 10% to 7% in the general population [1]. However, hepatitis B still remains endemic in China and other parts of Asia [2,3]. Blood screening strategies used in other developed countries with a reported low incidence of HBV infection include HBsAg and anti-HBc screening, and in some instances nucleic acid testing (NAT) [4-6]; whilst China relies almost solely on HBsAg serologic screening [7,8]. HBsAg mutations may give rise to changes in HBsAg antigenicity, resulting in reduced sensitivity and detection capability of current diagnostic tests, and which might present a challenge for HBsAg screening and HBV diagnosis, and ultimately increase the risk of transfusion-transmission of HBV infection [7-10].

In the absence of a proof-reading function, HBV reverse transcriptase randomly misincorporates bases into the replicating DNA strand, generating a quasispecies pool with a large number of variants [11]. Meanwhile, selection pressure from the specific immune response, whether from the passive application of hyperimmune globulin (HBIG) prophylaxis [12] or from active HBsAg vaccination [13,14], could drive the emergence of HBsAg mutant viruses. An amino acid change from glycine to arginine at position 145 (sG145R) for the immunodominant determinant of HBsAg, which alone can be responsible for vaccine escape, is most commonly reported and has been well documented [7,15]. A glycine to lysine point mutation at position 145 (sG145K) or a threonine to methionine substitution at position 118 (sT118M) has also been detected, and both mutations have been reported to significantly change the antigenic profile of HBsAg compared with that of wild type [7,16-18].

HBV mutants could also evolve because of selective pressure from nucleoside analog treatments. Lamivudine was the first approved oral nucleoside analogue for the treatment of chronic HBV infection, suppressing HBV replication by interfering with HBV DNA polymerase [19-21]. The substitution of methionine by isoleucine (I) or valine (V) in the tyrosine-methionine-aspartate-aspartate (YMDD) motif (C domain) at position 204 (rtM204I or rtM204V) has been shown to confer lamivudine resistance on HBV [22-24]. Interestingly, because of the overlap of the open reading frame (ORF) *S* for the HBsAg and ORF *P* for viral polymerase, rtM204I and rtM204V mutations produce sI195M and sW196S in the HBsAg [25,26]. Consequently, the YMDD mutation can occur naturally in chronic HBV infections in the absence of previous exposure to lamivudine treatment [27,28], highlighting the overlap of selective pressure between the immune response and drug treatment.

The double mutations may develop if the chronic HBV patients, who have been infected with immune-escaped mutant, receive anti-virus therapy or even in the absence of previous exposure to lamivudine treatment. Immune-escaped and drug-resistant mutants may also occur in some patients, who have been infected with drug-resistant mutants but been false negative in the HBsAg screening for the reduced antigenicity of the mutant S protein and receive hyperimmune globulin prophylaxis or HBsAg vaccination. Although substitutions outside of the ‘a’ determinant appear to be readily detected by current commercially available HBsAg immunoassays, there is limited information as to the combined effects of immune-escaped (T118M, G145K, G145R) and drug-resistant (rtM204I = sI195M, rtM204V = sW196S) point mutations on the antigenicity profiles of HBsAg. In the present study, we produced HBsAg double mutants (immune-escaped and drug-resistant) using site-directed mutagenesis and analyzed their binding capability using five commercially available ELISA kits in China.

## Results

### Expression of HBsAg mutants

**Figure 1:**
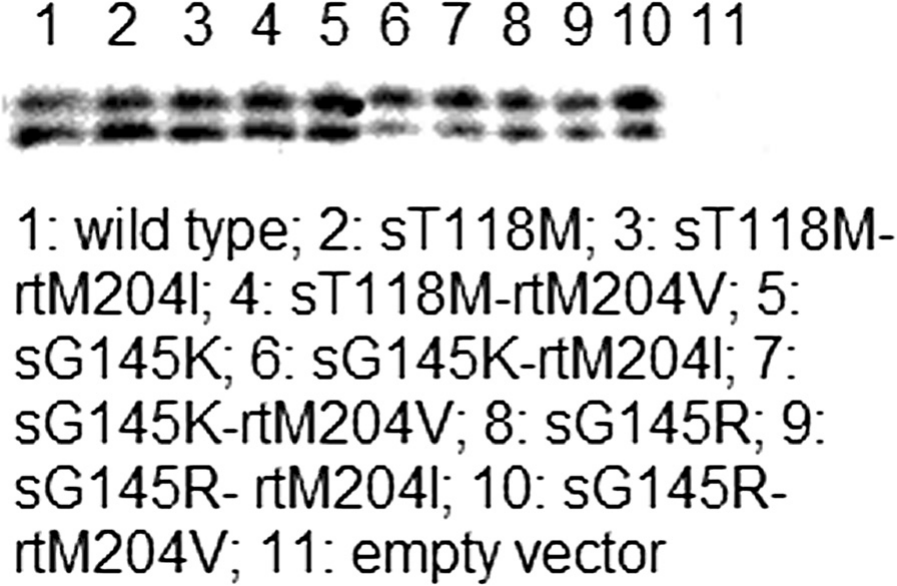
**Western blot analysis of the HBsAg expressed by wild type and mutant clones, plus empty vector as a negative control.** Transfected 293 T cell supernatant samples (10 μl/sample) were loaded to each lane in SDS-PAGE. HBsAg specific monoclonal antibody H166 was used as the detection antibody at 1:1000 dilution. 1: wild type; 2: sT118M; 3: sT118M-rtM204I; 4: sT118M-rtM204V; 5: sG145K; 6: sG145K-rtM204I; 7: sG145K-rtM204V; 8: sG145R; 9: sG145R- rtM204I; 10: sG145R- rtM204V; 11: empty vector.

To examine whether HBsAg mutants could express properly, 293 T cells were transfected transiently with each HBsAg mutant clone. With the wild-type HBsAg clone as the positive control and a mock DNA vector as the negative control, the expression of HBsAg mutants in 293 T cells was examined by Western blotting assay using monoclonal antibody H166, which recognized the amino acid 121–124 loop of HBsAg as a continuous epitope. These results indicated that 293 T cells transfected with either wild-type or HBsAg mutants had very comparable levels of HBsAg production (Figure 1). Since SDS denatured all proteins into linear shape, the overall antigenicity of proteins, including the configuration epitopes and linear epitopes, could not be loyally proved in the Western-blotting assay. Different from Western-blotting, ELISA is usually done to detect antigens in their native state, which reflects the antigenicity better.

### Negligible decline in the antigenicity of sT118M-rtM204I or sT118M-rtM204V mutant

**Figure 2:**
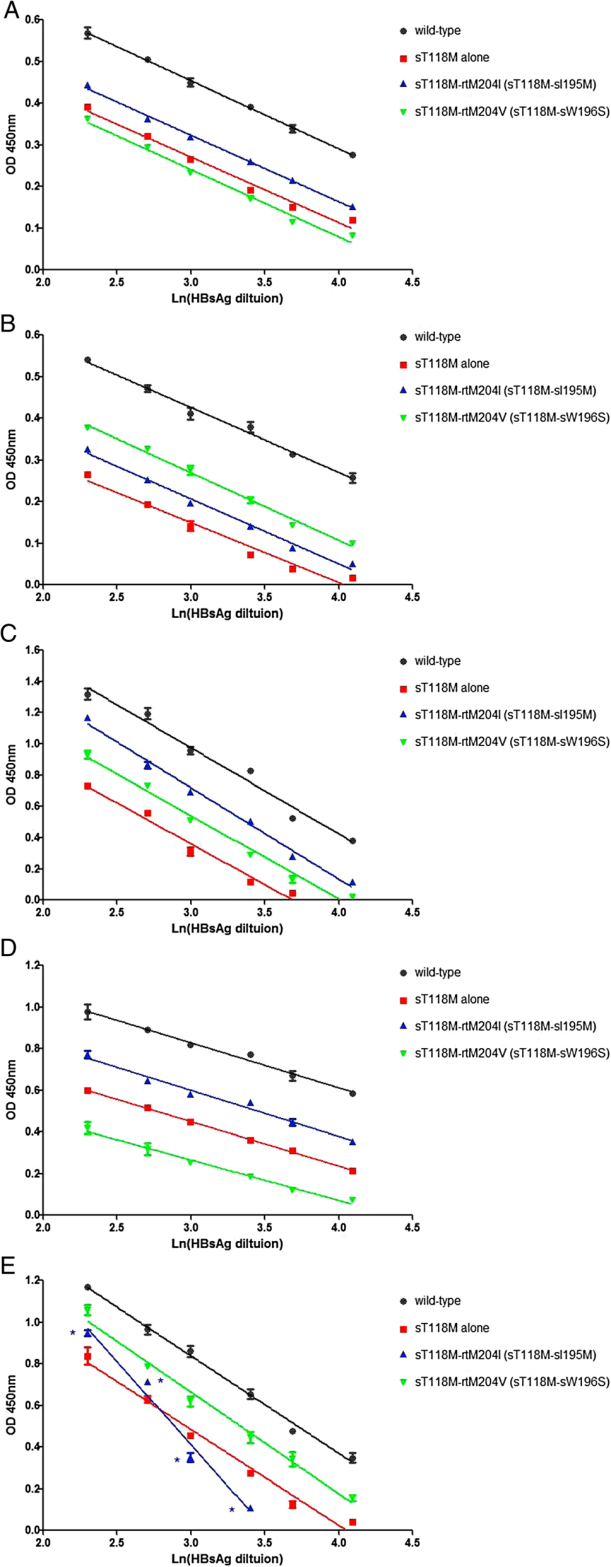
**The supernatants from each plasmid transfection were analyzed for HBsAg using five ELISA kits. (A)** Zhu Hai LivZon Diagnostic INC (LZ); **(B)** Beijing WanTai Biological Pharmacy Enterprise Limited Company (WT); **(C)** Beijing Big-GBI Biotech Limited Company (GBI); **(D)** Beijing BioNeovan Limited Company (BN); and **(E)** Shenzhen Kang Sheng Bao Bio-Technology Limited Company (KSB). With the exception of ELISA kit KSB, which recognized sT118M-rtM204I (sT118M-sI195M) with reduced avidity, all of the kits had almost invariable slope or avidity to the sT118M mutant, sT118M-rtM204I (sT118M-sI195M), and sT118M-rtM204V (sT118M-sW196S) double mutant. *, p < 0.05, compared with sT118M HBsAg.

Of the five commercial HBsAg ELISA kits used in this study, four kits (LZ, WT, GBT, and BN) recognized the sT118M immune-escaped mutant and recombinant sT118M-rtM204I (sT118M-sI195M) mutant, yielding similar titration curves, and indicating that rtM204I may contribute marginally to the antigenicity of sT118M HBsAg. Similarly, the differences in avidity (as determined by the slope of the curve) was indistinguishable between the sT118M mutant and sT118M-rtM204V (sT118M- sW196S) mutant for all five assays, suggesting that drug-resistant YMDD mutants caused a negligible loss in antigenicity for immune-escaped sT118M HBsAg (Figure 2).

### Significant reduction in the antigenicity of sG145K-rtM204I HBsAg mutant

**Figure 3:**
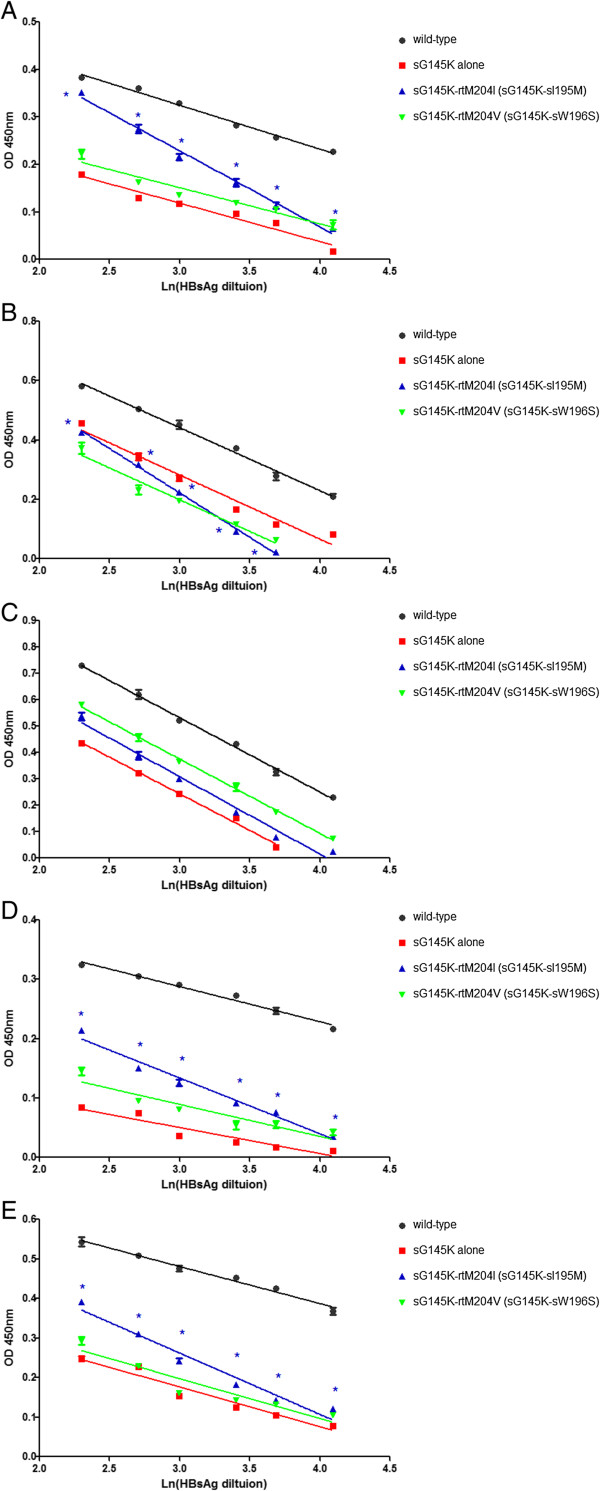
**The supernatants from each plasmid transfection were analyzed for HBsAg using five ELISA kits. (A)** Zhu Hai LivZon Diagnostic INC (LZ); **(B)** Beijing WanTai Biological Pharmacy Enterprise Limited Company (WT); **(C)** Beijing Big-GBI Biotech Limited Company (GBI); **(D)** Beijing BioNeovan Limited Company (BN); and **(E)** Shenzhen Kang Sheng Bao Bio-Technology Limited Company (KSB). With the rtM204I (sI195M) mutation in the backbone of sG145K, avidity was reduced significantly in four kits. The exception was with ELISA kit GBI. rtM204V (sW196S) mutation did not cause antigenic variation of sG145K HBsAg. *, p < 0.05, compared with sG145K HBsAg.

As with the recently identified T118M mutation in the ‘a’ determinant of HBsAg [7], the occurrence of sG145K or sG145R mutants are well reported [16-18], and they therefore may have a more significant role in infection diagnosis. With the rtM204I (sI195M) mutation on the backbone of the immune escape mutant sG145K, avidity was reduced significantly for four of the ELISA kits. The exception was with the GBI ELISA kit. These results suggest that unlike sT118M HBsAg, rtM204I (sI195M) may cause a considerable reduction in the antigenicity of sG145K mutant HBsAg. However, the rtM204V (sW196S) mutation, failed to cause a reduction in avidity in the five ELISA kits tested (Figure 3).

### Decrease in the antigenicity of sG145R-rtM204I/V HBsAg mutant

**Figure 4:**
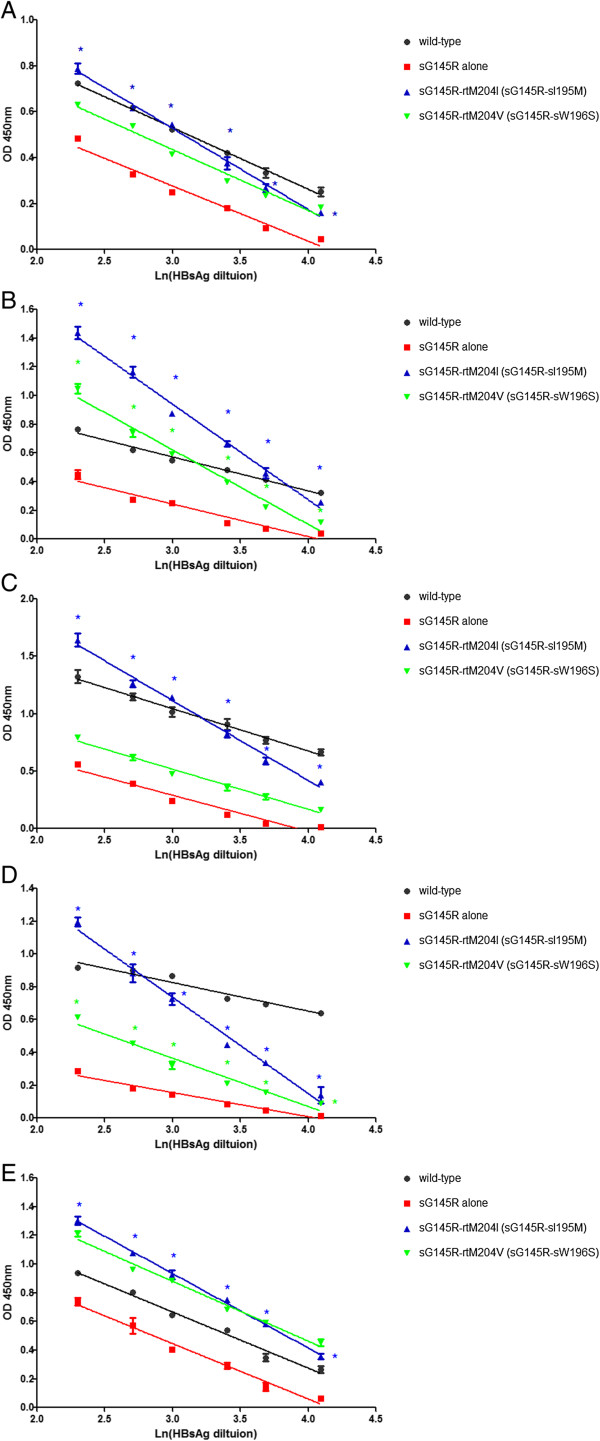
**The supernatants from each plasmid transfection were analyzed for HBsAg using five ELISA kits**. **(A)** Zhu Hai LivZon Diagnostic INC (LZ); **(B)** Beijing WanTai Biological Pharmacy Enterprise Limited Company (WT); **(C)** Beijing Big-GBI Biotech Limited Company (GBI); **(D)** Beijing BioNeovan Limited Company (BN); and **(E)** Shenzhen Kang Sheng Bao Bio-Technology Limited Company (KSB). With the rtM204I (sI195M) mutation in the backbone of sG145R, avidity was reduced significantly for all ELISA kits. rtM204V (sW196S) mutation also reduced the recognition of sG145R HBsAg in ELISA kits WT and BN. *, p < 0.05, compared with sG145R HBsAg.

The antigenicity profile also decreased for sG145R HBsAg when coupled with mutation rtM204I (sI195M) for all of the ELISA kits. Interestingly, rtM204V (sW196S) mutation also decreased the antigenicity of sG145R HBsAg but only for the WT and BN ELISA kits (Figure 4).

## Discussion

Currently, there are three lines of defense for HBsAg screening in transfusion centers across China. The first line of defense is the fast colloidal gold assay, which is performed prior to blood donation. Two rounds of screening using the more sensitive ELISA, using two commercially available kits from different companies, constitutes the two latter lines of defense [7]. Despite the current screening practices, post-blood transfusion HBV infection still persists [29,30], most probably due to the serologically negative window period (more so during the late stages of infection) [31], or through treatment [26]; all of which highlight the need for HBcAb screening and NAT in China. Unfortunately, HBcAb screening is hampered by the high prevalence of HBV infection in China. Although nationwide implementation of triplex-individual-donation-NAT testing for HBV DNA would detect an additional 9964 viremic donations per year in China [32], NAT is procedurally cumbersome and incurs high costs, and is therefore more suitable for developed countries with a low prevalence of HBV infection [33]. As HBsAg screening is fundamentally the only strategy which can be used to reduce post-transfusion HBV infection in China, the antigenicity of HBsAg, especially to mutants, should be explored further.

HBsAg mutants can evolve from either selective pressure from the immune response or through nucleoside analog treatments [34]. Although the 'a'-determinant region of HBsAg was usually unaffected by lamivudine therapy [35], mutations of amino acids outside the 'a'-determinant region could alter the antigenicity of HBsAg, especially considering their roles on the conformational changes of HBsAg. The unique gene structure of HBV dictates that variation within the shared coding region for HBsAg and virus polymerase may have an indirect affect on the other. This was demonstrated with the mutation sW196S in HBsAg, which was associated with reduced binding to anti-HBs antibody, and corresponding to rtM204I in polymerase and conferring resistance to lamivudine [36].

Regarding the co-existence of immune-escaped mutants and drug-resistant mutants, it is plausible to investigate their combined influence on the antigenicity of HBsAg. The immune escape mutant sT118M HBsAg can decrease the antigenicity of HBsAg considerably [37]. In the current study no changes to the antigenicity of sT118M HBsAg was observed when the drug-resistant mutants rtM204I (sI195M) or rtM204V (sW196S) were present on the sT118M backbone. YMDD mutations, particularly rtM204I (sI195M), reduced the antigenicity of sG145K HBsAg significantly, causing the great drop in the slope of an antibody titration curve in four of the five ELISA kits. The presence of rtM204I (sI195M) on the sG145R HBsAg mutant backbone, also resulted in a loss in avidity of ELISA kits or the antigenicity of HBsAg. Compared with rtM204I (sI195M), the YMDD mutation rtM204V (sW196S) appeared to have minimal influence on the immune-escaped HBsAg mutants. rtM204I (sI195M) significantly reduced the antigenicity of immune-escaped HBsAg, especially for the most common mutants sG145K and sG145R HBsAg. Of great concern is the possibility that escaped HBV mutants which may be not neutralized by antibodies induced by current HBsAg vaccines and unable to be detected using current HBsAg screening protocols, are being transmitted to other vaccinated individuals [26] who are blood donors in China.

## Conclusions

Drug-resistant YMDD mutations caused a significant reduction in the antigenicity of immune-escaped HBsAg, particularly for the most common mutant sG145K. The consequences of these results are that a reduction in HBsAg antigenicity may hinder HBsAg diagnosis and consequently increase the risk of HBV blood-transfusion transmission in China. To reduce the transmission of HBV through blood transfusions, HBsAg ELISA kits with greater analytic sensitivity and detection capability for wild type HBsAg and diverse mutants should be developed.

## Materials and methods

### Construction of plasmids encoding double mutant HBsAg by site-directed mutagenesis

Plasmid pJW4303 incorporating the optimized wild-type HBsAg gene (genotype B) was a gift from China-US Vaccine Research Center [38]. Site-directed mutagenesis of the HBsAg was achieved using the Altered Sites *in vitro* Mutagenesis Kit (Promega Corporation, Madison, WI.) according to the manufacturer’s instructions, and the primers listed in Table 1. Mutations were verified by sequencing prior to cloning into similarly digested empty parent vector pJW4303 to yield recombinant plasmids; pJW4303-sT118M-rtM204I, pJW4303-sT118M-rtM204V, pJW4303-sG145K-rtM204I, pJW4303-sG145K-rtM204V, pJW4303-sG145R-rtM204I, and pJW4303-sG145R-rtM204V. All recombinant plasmids used in transfection experiments were purified using Qiagen’s plasmid midi kit (Qiagen Inc., Chatsworth, CA.).

**Table 1:** **Primers used for site**-**directed mutagenesis**

**Primer**	**Sequence**
T118M-Forward	5’-TCATCAACAACCAGC***ATG***GGACCATGCAAA-3’
T118M-Backward	5’-TTTGCATGGTCC***CAT***GCTGGTTGTTGATGA-3’
G145K-Forward	5’-AAACCTACGGAC***AAA***AACTGCACCTGT-3’
G145K-Backward	5’-ACAGGTGCAGTT***TTT***GTCCGTAGGTTT-3’
G145R-Forward	5’-AAACCTACGGAC***CGA***AACTGCACCTGT-3’
G145R-Backward	5’-ACAGGTGCAGTT***TCG***GTCCGTAGGTTT-3’
rtM204I-Forward	5’-GAT***CCT***GATGATGTGGTACTGGG-3’
rtM204V-Forward	5’-GAT***GTG***GATGATGTGGTACTGGG-3’
rtM204M/I-Backward	5’-ACGGACAGCCACACGGTGGGGCTCA-3’

Table 1 Primers for site-directed mutagenesis.

### Transient transfection with the expression plasmids

Human embryonic kidney 293 T cells were maintained in high-glucose DMEM (Gibco) supplemented with 10% fetal calf serum (Hyclone) and 1 X Pen/Strep (Sigma) in 6-well tissue culture plates at 37°C and 5% CO_2_. Once the cells had reached 60 to 80% confluence they were transfected with recombinant plasmid DNA using PolyFect transfection reagent (Qiagen) according to a previously described method [39]. Briefly, 2 μg plasmid was mixed with 20 μl PolyFect transfection reagent and incubated at room temperature for 5–10 min before addition to 293 T cells. The culture supernatant of transfected cells was collected after 72 h and stored at −70°C until required for the detection of HBsAg.

### Western-blotting

To identify the expression of HBsAg construct, the culture supernatant from each HBsAg mutant transfected cells was subjected to SDS-PAGE and blotted onto PVDF membrane. Blocking was done with 0.1% I-Block (Tropix, Bedford, MA). The membrane was incubated with H166 anti-HBs from Abbott at 1:1000 dilution for 1 h and reacted subsequently with AP-conjugated goat anti-mouse IgG at 1:5000 dilution for 30 min. Membranes were washed with blocking buffer after each step. Western-light substrate was then applied to the membrane for 5 min. X-ray films were exposed to the membrane and developed by a Kodak processor.

### Elisa

The supernatants recovered from each transfection were serially diluted and analyzed for HBsAg using five of the most popular commercial ELISA kits in China; Zhu Hai LivZon Diagnostic INC (LZ), Beijing WanTai Biological Pharmacy Enterprise Limited Company (WT), Beijing Big-GBI Biotech Limited Company (GBI), Beijing BioNeovan Limited Company (BN), and Shenzhen Kang Sheng Bao Bio-Technology Limited Company (KSB). The ability of each of the ELISA to detect immune-escaped mutants (sT118M, sG145R, sG145K) had been confirmed in previous experiments (unpublished data). All assays were performed according to the manufacturer’s instructions.

### Statistical analysis

The slope of each titration curve, which is proportional to the average antibody avidity [40-42], was determined and compared for the statistical difference using the Fisher’s exact test.

### Ethics statement

Written informed consent was obtained from all study participants. Sera and plasma samples were collected from the study subjects. They were diagnosed with HBV infection. This study was carried out in strict accordance with the requirements for clinical studies established by the Nanjing Medical University, China. The protocol was approved by the Ethics Review Committee from the First Affiliated Hospital of Nanjing Medical University.

## Abbreviations

HBV: Hepatitis B virus; HBsAg: Hepatitis B surface antigen; ELISA: Enzyme-linked immunosorbent assay; NAT: Nucleic acid testing; PCR: Polymerase chain reaction; YMDD: Tyrosine-methionine-aspartate-aspartate; ORF: Open reading frame.

## Competing interests

The authors declare that they have no competing interests.

## Authors’ contributions

MZ drafted the manuscript. MZ, GG, and YY constructed HBsAg mutants. XC, QF and JC expressed the HBsAg protein and completed the ELISA. MZ, GG, and ZH designed the experiment and analyzed the data. All authors read and approved the final manuscript.

## Authors’ information

Mingshun Zhang and Guohong Ge: co-first authors.
